# Associations between gabapentinoids and suicidal behaviour, unintentional overdoses, injuries, road traffic incidents, and violent crime: population based cohort study in Sweden

**DOI:** 10.1136/bmj.l2147

**Published:** 2019-06-12

**Authors:** Yasmina Molero, Henrik Larsson, Brian M D’Onofrio, David J Sharp, Seena Fazel

**Affiliations:** 1 Department of Psychiatry, Warneford Hospital, University of Oxford, Oxford OX3 7JX, UK; 2 Department of Medical Epidemiology and Biostatistics, Karolinska Institutet, Stockholm, Sweden; 3 Centre for Psychiatry Research, Department of Clinical Neuroscience, Karolinska Institutet, Stockholm, Sweden; 4 School of Medical Sciences, Örebro University, Örebro, Sweden; 5 Department of Psychological and Brain Sciences, Indiana University, Bloomington, IN, USA; 6 Division of Brain Sciences, Imperial College London, London, UK

## Abstract

**Objective:**

To examine associations between gabapentinoids and adverse outcomes related to coordination disturbances (head or body injuries, or both and road traffic incidents or offences), mental health (suicidal behaviour, unintentional overdoses), and criminality.

**Design:**

Population based cohort study.

**Setting:**

High quality prescription, patient, death, and crime registers, Sweden.

**Participants:**

191 973 people from the Swedish Prescribed Drug Register who collected prescriptions for gabapentinoids (pregabalin or gabapentin) during 2006 to 2013.

**Main outcome measures:**

Primary outcomes were suicidal behaviour, unintentional overdoses, head/body injuries, road traffic incidents and offences, and arrests for violent crime. Stratified Cox proportional hazards regression was conducted comparing treatment periods with non-treatment periods within an individual. Participants served as their own control, thus accounting for time invariant factors (eg, genetic and historical factors), and reducing confounding by indication. Additional adjustments were made by age, sex, comorbidities, substance use, and use of other antiepileptics.

**Results:**

During the study period, 10 026 (5.2%) participants were treated for suicidal behaviour or died from suicide, 17 144 (8.9%) experienced an unintentional overdose, 12 070 (6.3%) had a road traffic incident or offence, 70 522 (36.7%) presented with head/body injuries, and 7984 (4.1%) were arrested for a violent crime. In within-individual analyses, gabapentinoid treatment was associated with increased hazards of suicidal behaviour and deaths from suicide (age adjusted hazard ratio 1.26, 95% confidence interval 1.20 to 1.32), unintentional overdoses (1.24, 1.19 to 1.28), head/body injuries (1.22, 1.19 to 1.25), and road traffic incidents and offences (1.13, 1.06 to 1.20). Associations with arrests for violent crime were less clear (1.04, 0.98 to 1.11). When the drugs were examined separately, pregabalin was associated with increased hazards of all outcomes, whereas gabapentin was associated with decreased or no statistically significant hazards. When stratifying on age, increased hazards of all outcomes were associated with participants aged 15 to 24 years.

**Conclusions:**

This study suggests that gabapentinoids are associated with an increased risk of suicidal behaviour, unintentional overdoses, head/body injuries, and road traffic incidents and offences. Pregabalin was associated with higher hazards of these outcomes than gabapentin.

## Introduction

Gabapentinoids have anticonvulsant, analgesic, and anxiolytic properties. The two main gabapentinoids, gabapentin and pregabalin, are currently approved for the treatment of epilepsy and neuropathic pain disorders in Europe (including Sweden). Pregabalin is also approved for treating generalised anxiety disorder in Europe, and has received approval for treating fibromyalgia in the United States.^1^ Prescriptions have risen steeply in recent years,^2^ and gabapentinoids are among the top 15 drugs globally in terms of revenue.^3^ ^4^ ^5^ Concerns have, however, been expressed about overprescription, particularly for pain relief,^2^ ^3^ as well as adverse effects,^6^ ^7^ including dizziness, somnolence, balance problems, blurred vision,^1^ ^6^ ^8^ ^9^ ^10^ ^11^ ^12^ coordination problems, and impairments in cognitive performance.^6^ ^13^ Although a 2008 Food and Drug Administration study of antiepileptic drugs reported an increased suicidal risk, separate analyses for pregabalin and gabapentin did not show clear effects.^14^ However, suicidal ideation accounted for most reported events; suicidal behaviour was rare.^14^ Since the FDA investigation, pharmacoepidemiological studies have used administrative data with larger sample sizes to examine suicidal outcomes for gabapentin.^15^ ^16^ Results have been inconsistent, with increased,^17^ decreased,^18^ and no changes in suicide risk.^19^ ^20^ ^21^ Furthermore, conclusions have been limited owing to confounding by indication^16^ (ie, the reason for prescribing the drug is also associated with the adverse outcome studied).

Gabapentinoids have been linked to overdoses and related deaths.^22^ In a nested case-control study of opioid users, concomitant gabapentin use was associated with a 60% increase in opioid related death compared with no concomitant use.^23^ In the United Kingdom, gabapentinoids are being reclassified as a class C controlled substance.^24^ ^25^ Evidence is, however, limited and is based on a small number of reported deaths.^26^ Little data exist on the association between gabapentinoids and other psychosocial adverse outcomes. Isolated cases of aggression have been reported for gabapentin in children with psychiatric morbidity.^27^ ^28^ ^29^ Most of this research is based on case reports, limiting conclusions about causality. Thus, evidence about adverse outcomes associated with gabapentinoids is limited, despite broadening clinical indications and increasing use.^6^ Furthermore, gabapentinoids are widely used off-label, which could account for up to 90% of prescriptions for pregabalin.^9^ Reliable information about adverse outcomes is required to test whether these concerns are valid, as inaccurate findings may influence prescribing practices.^13^ ^16^

To address these shortcomings, we applied a within-individual design to a large population based sample to examine associations between gabapentinoids and adverse outcomes related to coordination disturbances (head or body injuries, or both and road traffic incidents and offences), mental health (suicidal behaviour, unintentional overdoses), and criminality (violent crime).

## Methods

### Design

We carried out a population based cohort study using Swedish registers with national coverage, linked through unique identification numbers.^30^ A within-individual design was used, where participants acted as their own control, thus more fully accounting for confounding by indication and time invariant factors such as individual vulnerability or psychiatric history. Furthermore, we examined associations for specific subgroups based on sex, age, substance use disorders, use of other antiepileptic drugs, and pre-existing comorbidities to further clarify risks and benefits of gabapentinoids.

### Participants and setting

In the total population of Sweden aged 15 and older during the study period (n=8 945 712), we identified all those who had collected at least two consecutive prescriptions for gabapentinoids. Follow-up started on 1 January 2006 (or on the date of immigration to Sweden) and ended on 31 December 2013. Single prescriptions were not included in the main analyses owing to uncertainty over drug adherence or tolerance. We also collected demographic data (see Methods section in supplementary file). Our cohort included both prevalent users (ie, participants who used gabapentinoids before follow-up started) and new users.

### Drugs

All citizens in Sweden are insured through a common non-claims healthcare insurance, and drugs are subsidised. Our data consisted of dispensed drugs (filled prescriptions) from Swedish pharmacies, obtained from the Swedish Prescribed Drug Register. This register includes information on all prescriptions dispensed by pharmacies in Sweden since July 2005, with less than 0.3% missing information.^31^ We extracted information on prescriptions for pregabalin (Anatomical Therapeutic Chemical code N03AX16) and gabapentin (N03AX12) from the Swedish Prescribed Drug Register. The Swedish Pharmaceutical Benefits allows for a maximum of three months’ supply for each prescription.^32^ To ensure treatment continuity, we defined treatment periods as at least two consecutively collected prescriptions no more than three months apart. Participants were considered to have used the drugs from the date of the first prescription to the date of the last prescription within that treatment period (which could last from a few weeks to up to eight years). We considered prescriptions if they were dispensed more than three months apart to be the start of a new treatment period, and we investigated each treatment period in the analyses.^33^

### Outcomes

Information on suicidal behaviour, unintentional overdoses, head or body injuries, and road traffic incidents was collected from the Swedish Patient Register,^34^ which includes all admissions to hospitals in Sweden, as well as outpatient contacts with specialised secondary care. This register includes the primary diagnoses listed in 99% of all hospital discharges. In validation studies, the positive predictive value of diagnoses in this register is between 85% and 95%.^34^ Only diagnoses received during unplanned (emergency) visits were used in our analyses, and diagnoses received during planned visits (follow-ups and referrals) were excluded. Although this is a more conservative approach, we used this measure to avoid overestimation of the diagnoses, as the diagnosis that is the reason for treatment initiation could also be coded during follow-ups and referrals regardless of current symptoms. Information on death by suicide, unintentional overdoses, head or body injuries, and road traffic incidents was collected from the Cause of Death Register—a register of all deaths in Sweden; the underlying cause is specified in 96% of the cases.^35^ For violent crime, we used arrests as the primary outcome because some investigations may be dropped by the prosecution.^36^ Furthermore, the decision to discontinue criminal proceedings might or might not be influenced by the charged person taking psychotropic medication. In sensitivity analyses we used convictions (rather than arrests) as an outcome. We extracted information on suspected offences from the Register of People Suspected of Offences, including all those arrested of a crime after a completed investigation by police, customs authority, or prosecution service.^37^ Information on convicted offences came from the National Crime Register, including all convictions in Swedish district courts.^37^

*Suicidal behaviour*—suicidal behaviour was defined as emergency hospital visits due to self injurious behaviour or suicide attempt, or death by suicide (international classification of diseases, 10th revision (ICD-10) codes X60-X84).

*Unintentional overdoses*—unintentional overdoses were defined as emergency hospital visits or death due to poisoning by illicit drugs, medications, and biological substances (ICD-10: T36-T50), accidental poisoning by noxious substances (X40-X49), and acute intoxications and overdoses by alcohol and illicit drugs (F10.0, F11.0, F12.0, F13.0, F14.0), excluding intentional self poisoning (ICD-10: X60-X69).

*Head/body injuries*—head/body injuries were defined as emergency hospital visits or death due to superficial, open, or crushing injuries, dislocations, fractures, and amputations (ICD-10: S00-T14), with intentional self injuries (ICD-10: X70-X84) excluded. In sensitivity analyses, we stratified injuries into two separate categories; injuries to the head or neck (ICD-10: S00-S19) and injuries to the body (ICD-10: S20-T14).

*Road traffic incidents and offences*—road traffic incidents and offences were defined as emergency hospital visits or death due to road traffic accidents (ICD-10: V00-V99), or arrests or convictions of traffic offences (including reckless driving, hit and run offences, causing death or injury by driving, and moving violations, as in previous work^38^). In sensitivity analyses, we investigated hospital treatment for road traffic incidents and arrests or convictions for road traffic offences separately.

*Violent crime*—violent crime was defined as crimes against people, as in previous work,^39^ and included attempted, completed, and aggravated forms of murder, manslaughter, unlawful threats, harassment, robbery, arson, assault, assault on an official, kidnapping, stalking, coercion, and all sexual offences.

### Statistical analyses

All observable follow-up time was split into periods of treatment and non-treatment. We censored observations at the end of follow-up, or in the event of death or permanent emigration from Sweden. When a treatment period ended, the participant crossed over to a non-treatment period. If adverse outcomes were experienced during a period, this period was further split into the period before the first outcome, period between outcomes, and period after the last outcome. We measured time at risk from the start of all periods. To account for unobserved time—that is, periods where gabapentinoid use or adverse outcomes, or both might not have been captured in the registers—we removed (truncated) periods of intermittent emigration, prison stay, stay in secure residential homes for juveniles, and hospital admission. Time after immigration, hospital discharge, and release from prison or secure residential homes was added to the observed time again, and we measured time at risk from the start of this period.

We used a within-individual design, which was analysed by stratified Cox proportional hazards regression. This design is a variant of self controlled cases series, where participants serve as their own control.^40^ ^41^ In this design, the rate of adverse outcomes during all treatment periods is compared with the rate of adverse outcomes during all non-treatment periods within each participant. This reduces the potential for unmeasured confounding that is time invariant during the study period, such as due to genetics and historical factors. In the model, only those who change drug status contribute directly to the estimate. All others contribute indirectly through the estimate of the association with age. To adjust for age as a categorical time varying covariate, we coded age with one category for each whole year. We used restricted cubic splines to allow for non-linear effects of age.^42^ In sensitivity analyses, we stratified on predetermined age bands (15-24, 25-34, 35-44, 45-54, 55-64, and ≥65 years). Because the unadjusted covariates in the stratified Cox proportional hazards regression are time varying, we did not test for the proportional hazards assumption.

We initially analysed gabapentinoids as one class and examined associations for the whole sample. Then we analysed pregabalin and gabapentin separately. These samples were not mutually exclusive, as certain participants were dispensed both drugs during follow-up. Subsequent stratifications were made on sex and predetermined age bands.

Several additional analyses were carried out. To account for the potential effect of other antiepileptics, we excluded participants who had been dispensed another antiepileptic (ATC codes N03AA-AG, N03AX03-11, N03AX13-15, and N03AX17-30) during follow-up. To account for the influence of alcohol and drug use, we excluded all those with diagnosed substance use disorders (ICD-10: F10-F19, not including overdoses and acute intoxications F10.0, F11.0, F12.0, F13.0, F14.0). To examine if single prescriptions (ie, not part of a treatment period) were differently associated with adverse outcomes, we examined adverse outcomes 30 days after a single prescription was dispensed. We also carried out analyses where we examined participants who had collected only one gabapentinoid prescription during follow-up. In our main analyses, we used a more conservative measure to define the end of a treatment period—that is, we considered treatment to end at the date of the last prescription. To account for the possibility that participants take gabapentinoids for up to three months after their last dispensed prescription (as the Swedish Pharmaceutical Benefits allows for a maximum of three months’ supply for each prescription), we repeated the main models with a different definition of a treatment period, by extending the end of a treatment period to three months after the last collected prescription. To account for previous use of gabapentinoids, we included a two year wash-out period to include only those who had been treatment-free for at least 24 months before starting their first treatment during our follow-up (a new user design). To examine new onsets of adverse outcomes, we excluded those who had experienced an event of the examined outcome before starting gabapentinoid treatment.

To estimate dose of gabapentinoids used, we calculated the defined daily dose by summing dispensed drugs and then dividing the sum by the number of days in the treatment period. We then categorised defined daily doses into three separate treatment interval categories; low use (<1 defined daily dose), moderate use (1-2 defined daily doses), and high use (>2 defined daily doses), and we compared each treatment interval category to intervals with no use.

For all analyses, 95% confidence intervals are presented. We used SAS version 9.4 and STATA version 14.1. The Methods section in the supplementary file provides more information on statistical analyses, including sensitivity analyses. The strengthening the reporting of observational studies in epidemiology (STROBE) reporting guidelines were followed (see supplementary file).

### Sensitivity analyses

Separately, we analysed only participants who had a pre-existing comorbidity before the start of gabapentinoid treatment,^43^ ^44^ including epilepsy (n=10 891), psychiatric disorders (n=61 526), or musculoskeletal disorders (n=91 932) (ie, all approved indications for gabapentinoids in Sweden). We also carried out analyses where we excluded those with any of these pre-existing comorbidities (n=60 797). We studied long term associations by comparing all time before the first collected gabapentinoid to all time after, using a conditional fixed effects Poisson regression analysis. We tested if gabapentinoids were differentially associated with hospital treatment for road traffic incidents (when it is undetermined who caused the incident) and arrests or convictions for road traffic offences (which would be caused by the individual) by examining these separately. Similarly, we examined differential associations for head or neck injuries and body injuries separately. Finally, we examined convictions (as opposed to arrests) of violent crime.

### Patient and public involvement

No patients were involved in setting the research question or the outcome measures, nor were they involved in developing plans for recruitment, design, or implementation of the study. No patients were asked to advise on interpretation or writing up of results. There are no plans to disseminate the results of the research to study participants or the relevant patient community.

## Results

Overall 191 973 participants collected prescriptions of gabapentinoids on at least two consecutive occasions. Of those, 120 664 were dispensed pregabalin and 85 360 were dispensed gabapentin (n=14 051 were dispensed both drugs). In the gabapentinoid cohort, 59.1% (n=113 497) were women, and most of the sample was 45 years or older (table 1). During 2006 to 2013, 5.2% (n=10 026) of the treatment cohort presented to secondary medical services with suicidal behaviour or died from suicide, 8.9% (n=17 144) experienced an unintentional overdose, 6.3% (n=12 070) had a road traffic incident or offence, 36.7% (n=70 522) presented with head/body injuries, and 4.1% (n=7984) were arrested for a violent crime. Pregabalin users were younger and associated with a higher prevalence of all outcomes compared with gabapentin users (table 1).

**Table 1:** Sociodemographic and medical characteristics of participants prescribed and dispensed gabapentinoids. Values are numbers (percentages)

Characteristics	Gabapentinoid cohort (n=191 973)	Pregabalin cohort (n=120 664)*	Gabapentin cohort (n=85 360)*	Only one prescription collected (n=80 998)
Sex:				
Men	40.9 (78 476)	40.4 (48 796)	41.5 (35 457)	40.0 (32 037)
Women	59.1 (113 497)	59.6 (71 868)	58.5 (49 923)	60.0 (48 039)
Age at start of treatment (years):				
<25	3.7 (7117)	4.8 (5830)	1.8 (1574)	3.6 (2939)
25-34	8.1 (15 488)	10.4 (12 538)	4.6 (3910)	7.5 (6053)
35-44	12.8 (24 486)	15.2 (18 299)	9.4 (8008)	14.5 (11 764)
45-54	16.7 (32 115)	18.5 (22 343)	14.3 (12 235)	18.7 (15 109)
55-64	17.6 (33 805)	17.6 (21 239)	17.9 (15 302)	20.0 (16 202)
≥65	41.1 (78 962)	33.5 (40 415)	51.9 (44 331)	35.7 (28 931)
Occupation in 2006†:				
Employed	34.6 (66 469)	36.6 (44 123)	31.5 (26 864)	43.4 (33 619)
Student	4.6 (8840)	6.0 (7192)	2.5 (2089)	4.3 (3338)
Receiving state benefits‡	7.9 (15 230)	9.8 (11 869)	5.2 (4451)	7.7 (5943)
Receiving benefits owing to disability	24.7 (47 364)	26.4 (31 867)	23.5 (20 071)	22.0 (17 077)
Outcomes 2006-13:				
Suicidal behaviour and deaths from suicide	5.2 (10 026)	7.3 (8800)	2.5 (2091)	2.4 (1922)
Unintentional overdoses	8.9 (17 144)	11.7 (14 099)	5.4 (4605)	4.7 (3776)
Head/body injuries	36.7 (70 522)	37.8 (45 633)	35.9 (30 635)	32.5 (26 018)
Road traffic incidents and offences	6.3 (12 070)	7.3 (8828)	4.9 (4170)	5.7 (4524)
Convictions for violent crime	2.5 (4787)	3.8 (4070)	1.3 (1085)	1.7 (1347)
Arrests for violent crime	4.1 (7984)	5.6 (6763)	2.2 (1863)	2.9 (2283)
Health status before start of treatment:				
Epilepsy	5.7 (10 891)	5.4 (6483)	6.4 (5442)	2.5 (2009)
Psychiatric disorders	32.1 (61 526)	40.9 (49 384)	20.0 (17 031)	20.8 (16 808)
Musculoskeletal disorders	47.9 (91 932)	46.2 (55 714)	52.1 (45 340)	43.7 (35 375)
Concurrent drugs and substance use disorders 2006-13:				
Other antiepileptic drugs	17.4 (33 411)	19.8 (23 892)	15.0 (12 762)	9.7 (7858)
Substance use disorders	11.1 (21 344)	15.0 (18 038)	6.1 (5195)	7.7 (6249)

* Pregabalin and gabapentin cohorts are not mutually exclusive; 14 051 participants were dispensed both drugs during follow-up.

† Missing information on 54 070 participants.

‡ Housing and basic allowance for those below a certain income threshold.

**Fig 1:**
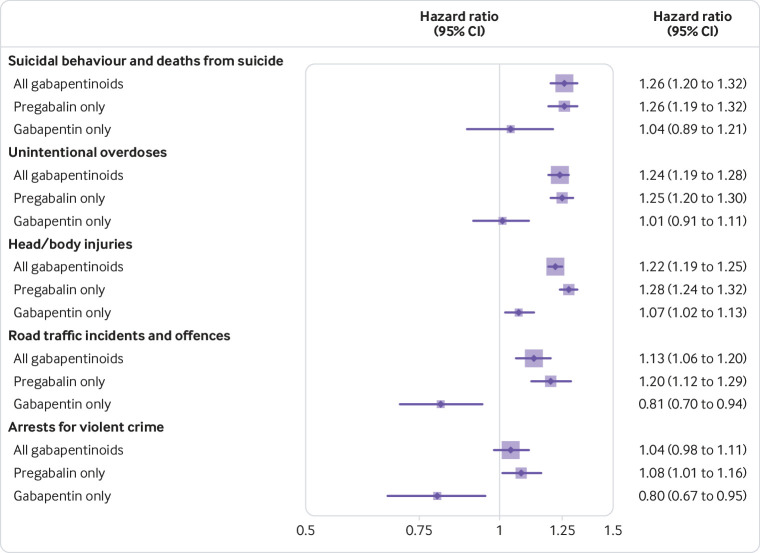
Within-individual associations between gabapentinoid treatment and adverse outcomes

In within-individual analyses, when treatment and non-treatment periods were compared in the same participant, gabapentinoids were associated with increased hazards of suicidal behaviour and deaths from suicide (age adjusted hazard ratio 1.26, 95% confidence interval 1.20 to 1.32), unintentional overdoses (1.24, 1.19 to 1.28), head/body injuries (1.22, 1.19 to 1.25), and road traffic incidents and offences (1.13, 1.06 to 1.20). Associations for arrests for violent crime were less clear (1.04, 0.98 to 1.11). When stratified on type of gabapentinoid, pregabalin was associated with increased hazards of all outcomes. In contrast, gabapentin treatment was associated with reductions in road traffic incidents and offences (0.81, 0.70 to 0.94) and arrests for violent crime (0.80, 0.67 to 0.95). No statistically significant associations were found for gabapentin and other outcomes (fig 1).

**Fig 2:**
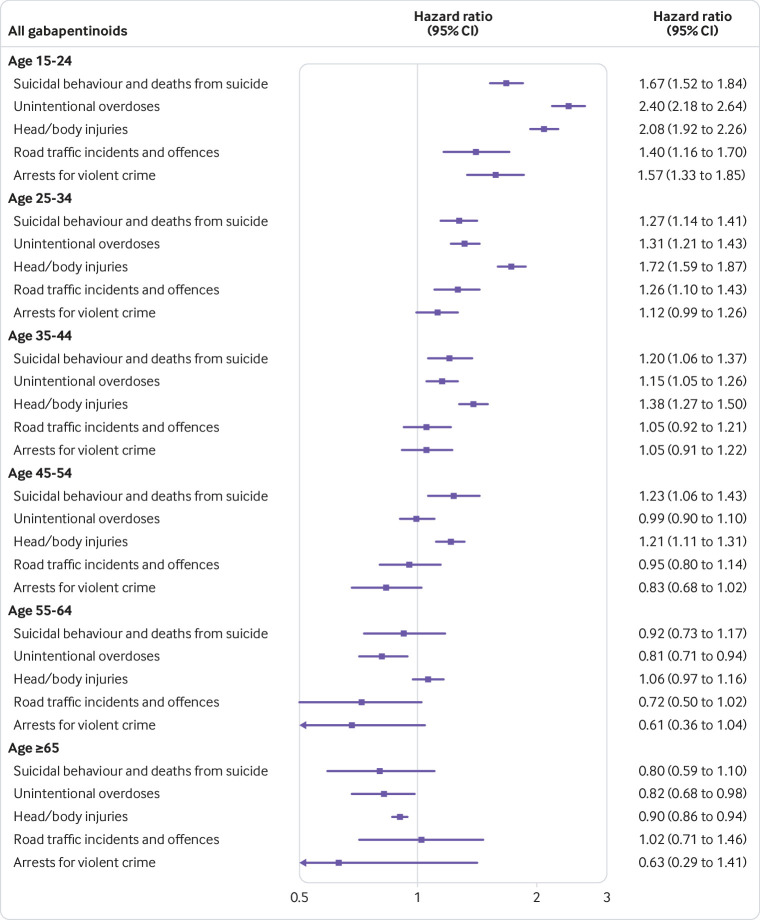
Within-individual associations between gabapentinoid treatment and adverse outcomes by age

By age band, results for gabapentinoids showed increased hazards of suicidal behaviour in people younger than age 55 and reduced or no associations in those aged 55 and older. The highest hazards were in the age group 15-24 years (1.67, 1.52 to 1.84). Patterns were similar for other outcomes; younger participants (15-34 years) showed increased hazards, whereas older participants (≥55 years) showed reduced or no associations (fig 2).

When drugs were examined separately, pregabalin treatment was associated with increases in all outcomes for younger participants (15-34 years), and suicidal behaviour, unintentional overdoses, and head/body injuries for those aged 35 to 44. Those aged 55 and older showed no or decreased associations with outcomes. Gabapentin treatment was associated with increased hazards of unintentional overdoses in participants younger than 25, and head/body injuries in participants aged 15-54. Reduced or no associations were found for participants aged 55 and older (see supplementary figures 1 and 2).

When participants who were prescribed another antiepileptic were excluded, the hazards increased for all outcomes except violent crime (table 2). After removing those with substance use disorders, we found attenuated associations between gabapentinoids and suicidal behaviour and head/body injuries, no statistically significant associations with unintentional overdoses and road traffic incidents and offences, and reduced associations with violent crime (table 2).

**Table 2:** Within-individual associations between gabapentinoid treatment and adverse outcomes, excluding participants who used other antiepileptics or with substance use disorders during follow-up

Adverse outcomes by excluded participants	Hazard ratio (95% CI)
Excluding those who used other antiepileptics during follow-up (n=158 562):	
Suicidal behaviour and deaths from suicide	1.30 (1.18 to 1.40)
Unintentional overdoses	1.24 (1.17 to 1.32)
Head/body injuries	1.13 (1.10 to 1.17)
Road traffic incidents and offences	1.10 (1.02 to 1.18)
Arrests for violent crime	1.01 (0.94 to 1.11)
Excluding those with substance use disorders during follow-up (n=170 629):	
Suicidal behaviour and deaths from suicide	1.14 (1.04 to 1.24)
Unintentional overdoses	0.99 (0.91 to 1.08)
Head/body injuries	1.09 (1.06 to 1.12)
Road traffic incidents and offences	0.94 (0.85 to 1.04)
Arrests for violent crime	0.83 (0.71 to 0.96)

We analysed 30 day treatment periods on the basis of single prescriptions, and found that single treatment periods were associated with increased hazards of suicidal behaviour, unintentional overdoses, and head/body injuries (table 3). Similarly, being dispensed only one prescription during follow-up was associated with increased hazards of these three outcomes.

**Table 3:** Within-individual associations between gabapentinoid treatment and adverse outcomes

Adverse outcomes by included participants	Hazard ratio (95% CI)
Single prescription treatment periods (n=102 363):	
Suicidal behaviour and deaths from suicide	4.40 (1.70 to 11.42)
Unintentional overdoses	4.94 (1.91 to 12.75)
Head/body injuries	2.27 (1.40 to 3.67)
Road traffic incidents and offences	1.33 (0.51 to 3.44)
Arrests for violent crime	1.20 (0.26 to 5.46)
Collected one prescription only (n=80 076):	
Suicidal behaviour and deaths from suicide	8.19 (1.58 to 42.42)
Unintentional overdoses	4.25 (1.19 to 15.15)
Head/body injuries	1.83 (1.07 to 3.13)
Road traffic incidents and offences	1.38 (0.43 to 4.38)
Arrests for violent crime	1.99 (0.18 to 22.00)
Adding three months after last collected prescription (n=191 973):	
Suicidal behaviour and deaths from suicide	1.36 (1.30 to 1.42)
Unintentional overdoses	1.31 (1.26 to 1.35)
Head/body injuries	1.25 (1.21 to 1.28)
Road traffic incidents and offences	1.18 (1.11 to 1.25)
Arrests for violent crime	1.12 (1.05 to 1.19)
Two year wash-out period (n=152 497):	
Suicidal behaviour and deaths from suicide	1.29 (1.23 to 1.36)
Unintentional overdoses	1.26 (1.21 to 1.31)
Head/body injuries	1.27 (1.23 to 1.30)
Road traffic incidents and offences	1.17 (1.10 to 1.25)
Arrests for violent crime	1.05 (0.99 to 1.12)
New onsets only:	
Suicidal behaviour and deaths from suicide (n=182 226)	5.13 (4.33 to 6.09)
Unintentional overdoses (n=176 278)	3.21 (2.89 to 3.57)
Head/body injuries (n=125 719)	4.19 (3.87 to 4.54)
Road traffic incidents and offences (n=172 766)	7.31 (5.57 to 9.59)
Arrests for violent crime (n=184 114)	3.79 (2.98 to 4.81)

We accounted for late treatment effects by extending the end of a treatment period to three months after the last collected prescription. Results showed increased associations with all outcomes, including violent crime. We included a two year wash-out period (a new user design) by including only those who had been treatment-free for at least 24 months before starting their first treatment during the study follow-up. These analyses showed increased associations with all outcomes except for violent crime, and hazards remained similar to those of the main analyses (table 3). Furthermore, we excluded those with a past event of the examined outcome before starting gabapentinoid treatment to examine new onset adverse outcomes. Hazards increased for all outcomes, ranging between 3.21 (95% confidence interval 2.89 to 3.57) for unintentional overdoses to 7.31 (5.57 to 9.59) for road traffic incidents and offences.

Finally, we examined associations between gabapentinoid dose and outcomes (table 4). For suicidal behaviour and unintentional overdoses, low and moderate doses were associated with similar hazards and high doses were associated with increased hazard ratios. For head/body injuries and road traffic incidents and offences, hazard ratios increased with use of higher dose. No clear links were found between dose and violent crime arrests.

**Table 4:** Within-individual associations between gabapentinoid treatment and adverse outcomes in individuals treated with gabapentinoids stratified by defined daily dose (DDD)

Adverse outcomes	Low use (<1 DDD)	Moderate use (1-2 DDDs)	High use (>2 DDDs)
Suicidal behaviour and deaths from suicide	1.33 (1.26 to 1.41)	1.31 (1.22 to 1.40)	1.38 (1.27 to 1.50)
Unintentional overdoses	1.25 (1.20 to 1.30)	1.25 (1.18 to 1.32)	1.39 (1.30 to 1.48)
Head/body injuries	1.17 (1.14 to 1.20)	1.28 (1.23 to 1.34)	1.42 (1.34 to 1.50)
Road traffic incidents and offences	1.15 (1.06 to 1.25)	1.21 (1.09 to 1.34)	1.32 (1.19 to 1.47)
Arrests for violent crime	1.10 (1.01 to 1.20)	1.06 (0.96 to 1.18)	1.24 (1.12 to 1.37)

Reference category was periods with no use.

Number of intervals with no use (16 071 061, 81.8%), low use (2 288 928, 11.7%), moderate use (788 068, 4.0%), and high use (496 935, 2.5%).

### Sensitivity analyses

On stratification by sex, results for men and women mostly followed the same patterns as for the overall analyses (see supplementary table 1).

We analysed participants who had a diagnosis of comorbid epilepsy, psychiatric disorders, or musculoskeletal disorders before the start of gabapentinoid treatment (see supplementary table 2). In comorbid epilepsy, gabapentinoids were associated with reduced hazards for all outcomes apart from suicidal behaviour. In comorbid psychiatric disorders, gabapentinoids were associated with lower risk for all outcomes. In comorbid musculoskeletal disorders, gabapentinoids were associated with reductions in head/body injuries, road traffic incidents and offences, and arrests for violent crime. When participants without any of these pre-existing comorbidities were examined, the hazards associated with suicidal behaviour and overdoses increased and the hazards associated with head/body injuries decreased.

We examined long term associations and found that compared with time before the first dispensed gabapentinoid, time after was associated with higher incidence rate ratios of all outcomes, apart from road traffic incidents and offences (see supplementary table 3). When traffic incidents and offences were analysed separately, gabapentin was associated with decreased hazards of road traffic offences, whereas pregabalin was associated with increased hazards of both road traffic incidents and offences. When stratifying on type of injury, gabapentinoids were associated with both head or neck and body injuries. No statistically significant associations for convictions for violent crime were shown (see supplementary table 4).

## Discussion

Gabapentinoid treatment in those aged 55 and older was associated with decreased hazards or no clear associations with suicidal behaviour, unintentional overdoses, head/body injuries, road traffic incidents or offences, and arrests for violent crime. Associations were, however, increased for all outcomes among the youngest cohort members (15-24 years). Participants in the other age bands showed heterogeneous associations, with increased hazards of suicidal behaviour, unintentional overdoses, and head/body injuries, and no associations with road traffic incidents or offences and arrests for violent crimes. When analysing gabapentinoids separately, pregabalin use was associated with increased hazards of all outcomes, whereas there were decreased or no associations for gabapentin.

### Strengths and limitations of this study

The strengths of this study include a large population based cohort, examination of a wide range of outcomes, inclusion of clinical outcomes from high quality nationwide registers, and comprehensive information on gabapentinoid treatment, as each collected prescription is registered. By using a within-individual design, we could adjust for time invariant covariates and more fully deal with unobserved confounders such as confounding by indication. This report does, however, have several limitations. As we principally examined associations, caution needs to be exercised when drawing causal inferences. We also lacked information on time varying covariates, such as alcohol or drug use, that could modify associations. To address this, we performed analyses where we excluded participants with substance use disorders. Furthermore, we did not have information on treatment adherence, which is a similar limitation in clinical trials, although this study is an improvement on studies that use solely prescription data as we had information on dispensed (ie, collected) drugs. To account for this, we excluded those who collected single prescriptions.

Results could further be affected by a potential bias towards the null owing to misclassification of gabapentinoid use. Our measure on treatment discontinuation was based on a more conservative assumption; the date of the last collected prescription. This assumption could result in slightly lower sensitivity, thus underestimating associations. We did, however, also carry out analyses where we extended the end of a treatment period to three months after the last collected prescription, and these results showed a clearer association with violent crime.

The use of register data might involve selection effects and potentially underestimate rates of underlying disorders and outcomes but at the same time it captures information with associated healthcare and other costs. We did not have information on indications for gabapentinoids, as this is not specified in the Swedish Prescribed Drug Register. Furthermore, this register started in July 2005, and previous dispenses are not recorded. Although we excluded treatment periods from July 2005 to 1 January 2006 (start of follow-up), participants could have been treated with gabapentinoids before that time. We did, however, include a wash-out period of two years. That is, in separate analyses we examined only those who had been treatment-free for at least 24 months before starting their first treatment during our follow-up. Results from these analyses were similar to the overall findings.

Finally, differences in prescription practices and outcome rates might affect the generalisability of findings. Sweden and the UK reported the lowest rates in Europe of road traffic incidents during the study period. While the UK had median levels of fatal falls among older adults, the rate in Sweden was among the highest in Europe.^45^ ^46^ Sweden and the UK are among the countries with the highest rates of drug induced mortality (50 to 60 per million population during the study period); however, rates have increased more steeply in Sweden in recent years.^47^ Sweden reports above both global and UK average rates for suicide mortality but below the regional rate (average rates per 100 000: 14.8 in Sweden, 8.9 in the UK, 15.4 in Europe, and 10.6 globally).^48^ During the study period, pregabalin was among the most commonly prescribed drugs in Sweden and the UK, as well as globally.^49^ ^50^

### Relation to previous studies

Previous research on gabapentinoids and suicidal behaviour has been inconsistent because of differing definitions, methods, and the extent of adjustment for confounding. Our use of a within-individual design allowed for further adjustments to handle confounding by indication. In our study, pregabalin but not gabapentin was associated with an increased hazard of suicidal behaviour. The differential results of the two gabapentinoids could be due to their different pharmacodynamic and pharmacokinetic profiles^1^; pregabalin has a higher potency, greater bioavailability, and quicker absorption than gabapentin.^8^ ^51^ ^52^ Pregabalin has also been associated with withdrawal symptoms following rapid discontinuation, which could be related to suicidal behaviour.^9^ ^11^ ^53^ ^54^ Several reports about gabapentinoid related overdoses and deaths have appeared.^3^ ^11^ ^22^ ^24^ ^52^ ^54^ ^55^ ^56^ ^57^ ^58^ Notably, when excluding participants with substance use disorders from our analyses, associations with suicidal behaviour were attenuated, and no associations remained for overdoses. This could suggest that simultaneous substance use increases the risk. This is in line with research showing that the prevalence of gabapentinoid misuse is higher among people who misuse opioids,^57^ and that overdoses of gabapentinoids are associated with respiratory depression and cardiac insufficiency if combined with sedatives or opioids.^23^ ^58^

Pregabalin and gabapentin also showed contrasting associations with road traffic incidents and offences and violent crime; gabapentin was associated with decreased hazards of these outcomes, whereas pregabalin was associated with increases. These differences are in keeping with the motor disturbances observed in clinical trials for pregabalin, but not for gabapentin.^6^ ^13^ Variations in the use of pregabalin and gabapentin (eg, off-label prescription, concomitant alcohol or drug use, or other time varying covariates) could also explain these differences. Furthermore, when we excluded participants with substance use disorders, we found no associations with unintentional overdoses and road traffic incidents and offences, which is in line with the suggestion that concurrent use of illicit drugs (particularly opioids) increases the risk of overdoses.^23^ A dose related effect has been suggested for pregabalin, with increases in adverse outcomes for larger doses.^9^ Our results were broadly consistent with a dose-response relation, although low doses of gabapentinoids were also associated with adverse events. Moreover, gabapentinoids were associated with greater hazards when we excluded participants who had experienced an adverse event (eg, unintentional overdose) before starting gabapentinoid treatment, suggesting stronger associations with new onsets of adverse outcomes.

Associations with adverse outcomes were mainly shown in those aged 15 to 24 years. Participants within this age band consistently presented increased rates of all adverse outcomes across several sensitivity analyses, particularly for suicidal behaviour, unintentional overdoses, and head/body injuries. This may be an age related pharmacodynamic effect, as has been suggested for selective serotonin reuptake inhibitors.^33^ ^59^ Young people have faster metabolism, which could lead to withdrawal problems, affecting impulsivity and emotion.^60^ Previous studies show that young people more often experience psychotropic drug induced behavioural difficulties, including hyperactivity and aggression.^13^ ^60^ Associations in young people might also be mediated by impulsivity and risk taking, or by different indications for their use. The increased associations with adverse outcomes among 15 to 24 year olds could also suggest that young people use alcohol or illicit drugs together with gabapentinoids, and that this combination increases the risk of adverse outcomes. Notably, the strongest association for unintentional overdoses was shown among young people, and associations decreased with age. Because of the suggested increased risk of overdoses when combining gabapentinoids with other substances,^23^ ^57^ our results could suggest that younger people use gabapentinoids with alcohol or drugs to a greater extent. The reduced hazards in older people could reflect pharmacodynamic differences related to age,^61^ ^62^ less concurrent use of alcohol or drugs, different indications for treatment, or reduced symptom severity of underlying conditions. These age related differences warrant further examinations across new samples and designs. Similarly, more research is needed to identify possible risk factors that mediate or moderate associations with adverse outcomes in younger people, as well as head-to-head comparisons between pregabalin and gabapentin.

### Conclusions

We found that gabapentinoids were associated with both increased and decreased risks of important adverse outcomes. These associations varied with age and type of gabapentinoid. Overall, gabapentinoids seem to be safe for a range of outcomes in older people. However, the increased risks found in adolescents and young adults (ages 15-24 years) prescribed gabapentinoids, particularly for suicidal behaviour and unintentional overdoses, warrant further research. If our findings are triangulated with other forms of evidence, clinical guidelines may need review regarding prescripgtions for young people, and those with substance use disorders. Further restrictions for off-label prescription may need consideration.

**What is already known on this topic**

Studies have linked gabapentinoids to suicidal behaviour and overdose related deaths
Research has been inconsistent and conclusions have been limited by methodological problems
Evidence about associations between gabapentinoids and other adverse outcomes is limited

**What this study adds**

This study suggests that gabapentinoids were associated with increased risks of suicidal behaviour, unintentional overdoses, head/body injuries, and road traffic incidents and offences
Pregabalin was associated with higher hazards of these outcomes than gabapentin, and these associations were strongest in those aged 15 to 24, where hazards were increased for all outcomes
Guidelines for gabapentinoid treatment in young people may need review

## Web extra

Extra material supplied by authors

## Notes

Contributors: YM and SF conceived and designed the study with input from HL, BDM, and DJS. YM analysed the data. YM had full access to all the data and carried out the data analyses. YM and SF wrote the first draft of the paper. All authors interpreted the data and contributed to the writing of the paper. All authors revised and approved the final version. YM and SF act as guarantors. The corresponding author attests that all listed authors meet authorship criteria and that no others meeting the criteria have been omitted.

Ethical approval: This study was approved by the regional ethics review board in Stockholm, Sweden (2013/5:8) and follows the Declaration of Helsinki.

Data sharing: The Public Access to Information and Secrecy Act in Sweden prohibits us from making individual level data publicly available. Researchers who are interested in replicating our work can apply for individual level data at Statistics Sweden: www.scb.se/en/services/guidance-for-researchers-and-universities/ .

Transparency The lead authors (YM and SF) affirm that this manuscript is an honest, accurate, and transparent account of the study being reported; that no important aspects of the study have been omitted; and that any discrepancies from the study as planned (and, if relevant, registered) have been explained.
