# Supplementary material for: Associations between gabapentinoids and suicidal behaviour, unintentional overdoses, injuries, road traffic incidents, and violent crime: population based cohort study in Sweden
Source: BMJ. 2019 Jun 12;365:l2147. doi: 10.1136/bmj.l2147 (PMC6559335; doi:10.1136/bmj.l2147)
Supplement: Supplementary file 1 — Supplementary information: additional methods, tables, and figures [file moly046157.ww1.pdf]

SUPPLEMENTAL METHODS

Demographic data

Information on age was collected from the Total Population Register[1], maintained by Statistics Sweden. Information on occupation was collected from the Longitudinal Integration Database for Health Insurance and Labour Market Studies, including annual data on all individuals aged 16 years and older residing in Sweden[2]. Information on migration was collected from the Migration Register[1], and information on prison and secure residential homes for juveniles was identified in the Prison Register[3].

Sensitivity analyses

We carried out separate analyses on pre-existing comorbidity, including only individuals who had received a diagnosis (during hospital visits or outpatient contacts with specialised secondary care) before the start of gabapentinoid treatment, belonging to one of the three categories of approved indications for gabapentinoid treatment in Sweden[4 5]. This included pre-existing epilepsy (ICD-10: F44.5, F80.3, G40-G41, P90, R56; ICD-9: 300.11, 333.2, 345, 649.4, 779.0, 780.3)[6] (n=10,891), pre-existing psychiatric disorders (ICD-10: F20-F43, F44.1-F44.4, F44.6-F44.9, F45-F48F50-F69, F90-F98; ICD-9: 295-299, 300.0, 300.2-300.9, 301-302, 307-316) (n=61,526), and pre-existing musculoskeletal disorders (ICD-10: M00-M68) (n=91,932). We also carried out analyses that excluded all individuals with these comorbidities before the start of gabapentinoid treatment (n=60,797).

To study long-lasting effects of gabapentinoid treatment, we compared all time before the start of the first collected gabapentinoid to all time after during the follow-up period. For this, we carried out an alternative within-individual analysis – using the conditional fixed-effects Poisson regression analysis – where the number of adverse events before the start of the first collected gabapentinoid were compared to the number of adverse events after the start.

REFERENCES

1. Ludvigsson JF, Almqvist C, Bonamy AK, et al. Registers of the Swedish total population and their use in medical research. *Eur J Epidemiol* 2016;31(2):125-36.
2. Statistics Sweden [Statistiska Centralbyrån]. Background Facts, Labour and Education Statistics 2011:4, Integrated database for labour market research [Longitudinell integrationsdatabas för Sjukförsäkrings- och Arbetsmarknadsstudier (LISA) 1990-2013, 2011:4]. Örebro: SCB, Bakgrundsfakta 2016:1 ISSN 1654-465X, 2016.
3. The Swedish Prison and Probation Service [Kriminalvården]. PPSS 2015: Prison and Probation Service and Statistics [KOS 2015: Kriminalvård och statistik]. Norrköping: The Swedish Prison and Probation Service [Kriminalvården] ISSN 1400-2167 2015.
4. Pregabalin. In Fass.se. http://www.fass.se/LIF/atcregister?userType=0, 2017.
5. Gabapentin. In Fass.se. http://www.fass.se/LIF/atcregister?userType=0, 2017.
6. Jette N, Beghi E, Hesdorffer D, et al. ICD coding for epilepsy: Past, present, and future—A report by the International League Against Epilepsy Task Force on ICD codes in epilepsy. *Epilepsia* 2015;56(3):348-55.

Supplemental Figure 1. Within-individual associations of pregabalin treatment and adverse outcomes by age (n=120,664).

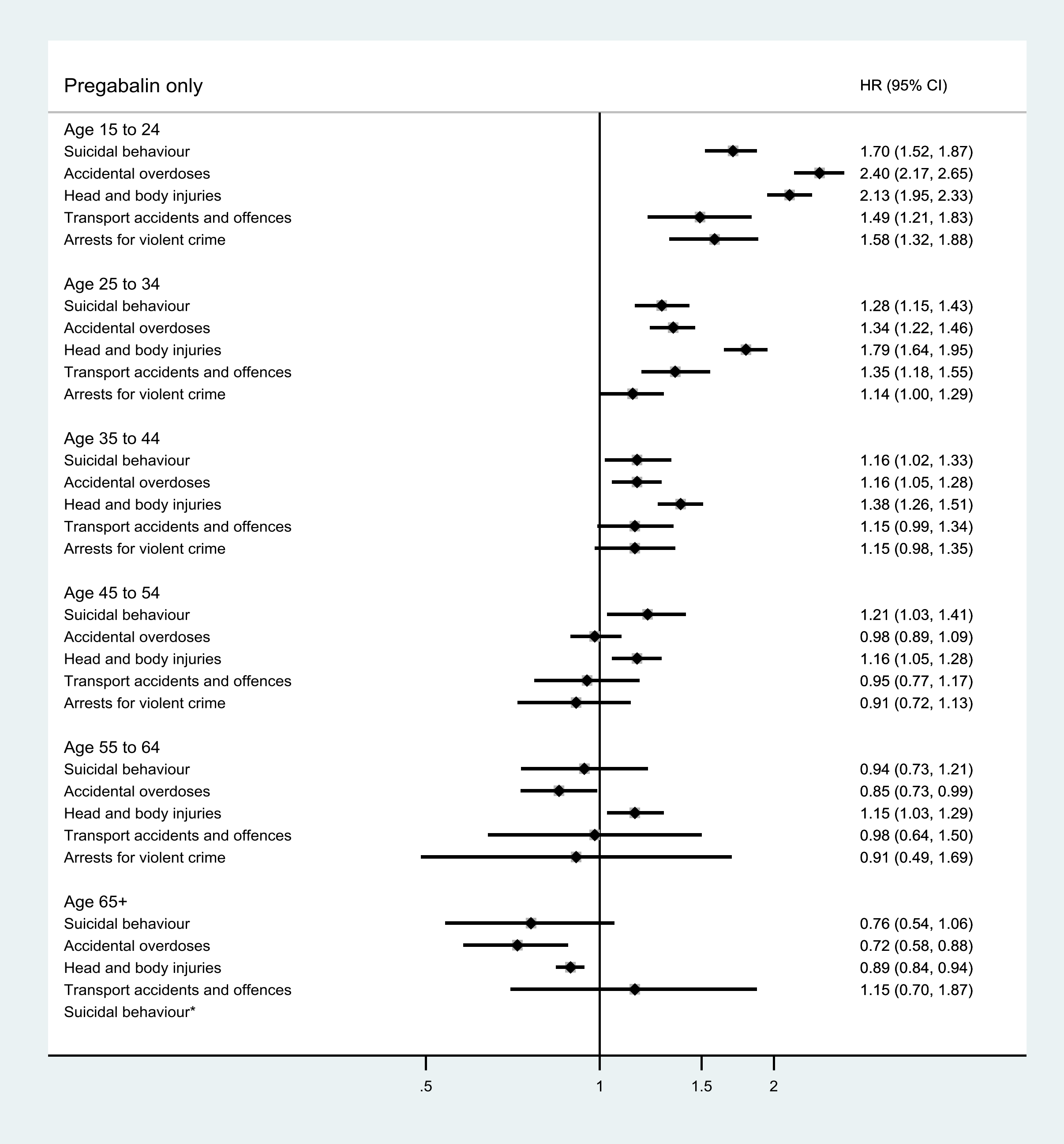

Note: Individuals under age 15 (i.e. the age of criminal responsibility in Sweden) were not included in the age-band analyses.
Number of events are reported in Suppl. Table 9.
* Too few individuals who changed medication status and/or presented with an event to allow for age-band specific analyses.

Supplemental Figure 2. Within-individual associations of gabapentin treatment and adverse outcomes by age (n=85,360).

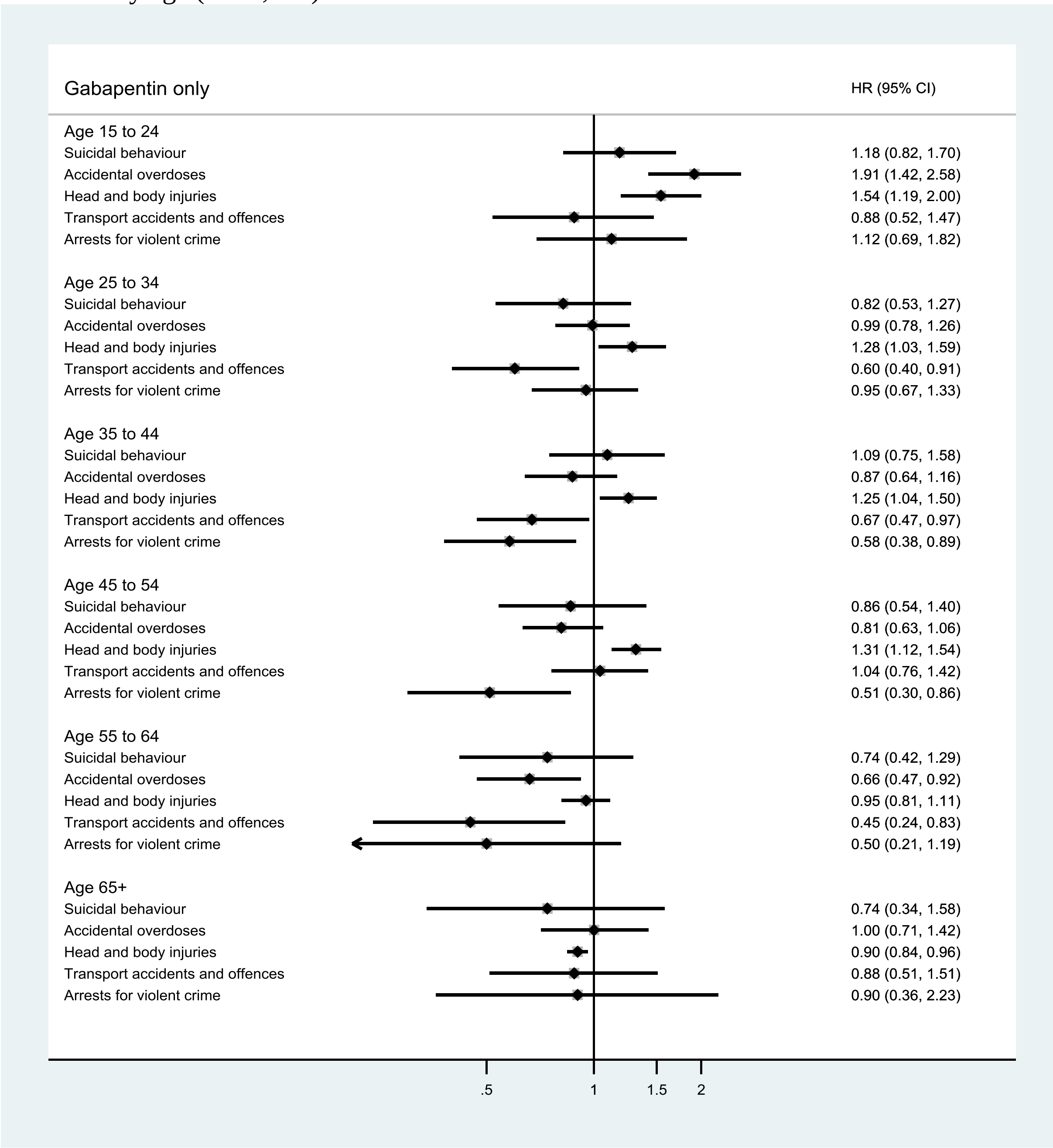

Note: Individuals under age 15 (i.e. the age of criminal responsibility in Sweden) were not included in the age-band analyses.
Number of events are reported in Suppl. Table 9.

Supplemental Table 1. Within-individual associations of gabapentinoid treatment and adverse outcomes by sex.

	**Within-individual analyses – Men HR (95% CI)**	**No. of events**	**Within-individual analyses – Women HR (95% CI)**	**No. of events**
**All gabapentinoids (n=191,973)**				
Suicidal behavior	1.44 (1.32 to 1.58)	6,452	1.21 (1.14 to 1.28)	12,907
Accidental overdoses	1.23 (1.17 to 1.30)	16,060	1.29 (1.22 to 1.36)	17,148
Head and body injuries	1.29 (1.19 to 1.41)	43,090	1.19 (1.11 to 1.27)	60,559
Transport accidents and offences	1.10 (1.03 to 1.18)	14,144	1.36 (1.20 to 1.55)	5,839
Arrests for violent crime	1.05 (0.98 to 1.13)	12,285	1.11 (0.95 to 1.30)	2,771
**Pregabalin only (n=120,664)**				
Suicidal behavior	1.40 (1.27 to 1.54)	5,634	1.22 (1.15 to 1.29)	11,749
Accidental overdoses	1.24 (1.17 to 1.31)	13,303	1.31 (1.24 to 1.38)	14,869
Head and body injuries	1.41 (1.26 to 1.56)	28,685	1.29 (1.19 to 1.40)	39,201
Transport accidents and offences	1.18 (1.09 to 1.27)	11,035	1.40 (0.80 to 1.20)	4,160
Arrests for violent crime	1.10 (1.02 to 1.18)	10,279	1.14 (0.96 to 1.35)	2,312
**Gabapentin only (n=85,360)**				
Suicidal behavior	1.27 (0.98 to 1.66)	1,245	0.96 (0.79 to 1.16)	1,972
Accidental overdoses	1.08 (0.94 to 1.23)	4,005	0.94 (0.81 to 1.10)	3,386
Head and body injuries	1.12 (0.97 to 1.30)	16,938	0.97 (0.87 to 1.09)	24,373
Transport accidents and offences	0.70 (0.58 to 0.85)	3,896	1.24 (0.98 to 1.57)	2,029
Arrests for violent crime	0.81 (0.67 to 0.98)	2,744	0.86 (0.58 to 1.28)	653

Supplemental Table 2. Sensitivity analyses: Within-individual associations of gabapentinoid treatment and adverse outcomes by pre-existing comorbidities

	**Within-individual analyses HR (95% CI)**	**No of events**
**Individuals with pre-existing comorbid epilepsy (n=10,891)**		
Suicidal behavior	0.96 (0.83 to 1.11)	3,034
Accidental overdoses	0.86 (0.78 to 0.94)	6,505
Head and body injuries	0.76 (0.64 to 0.89)	3,879
Transport accidents and offences	0.81 (0.66 to 0.99)	2,166
Arrests for violent crime	0.75 (0.62 to 0.91)	2,084
**Individuals with pre-existing comorbid psychiatric disorders (n=61,526)**		
Suicidal behavior	0.92 (0.87 to 0.98)	17,891
Accidental overdoses	0.91 (0.87 to 0.96)	27,604
Head and body injuries	0.79 (0.72 to 0.87)	12,889
Transport accidents and offences	0.89 (0.81 to 0.97)	11,255
Arrests for violent crime	0.81 (0.75 to 0.89)	10,907
**Individuals with pre-existing comorbid musculoskeletal disorders (n=91,932)**		
Suicidal behavior	0.90 (0.81 to 1.01)	6,223
Accidental overdoses	0.93 (0.86 to 1.01)	12,301
Head and body injuries	0.71 (0.65 to 0.75)	18,765
Transport accidents and offences	0.81 (0.72 to 0.91)	8,832
Arrests for violent crime	0.83 (0.72 to 0.95)	8,832
**Individuals with no pre-existing history of comorbidities (n=60,797)**		
Suicidal behavior	1.59 (1.19 to 2.13)	781
Accidental overdoses	1.46 (1.25 to 1.71)	2,536
Head and body injuries	0.84 (0.75 to 0.94)	21,710
Transport accidents and offences	0.95 (0.83 to 1.09)	4,199
Arrests for violent crime	1.08 (0.93 to 1.27)	2,430

Supplemental Table 3. Sensitivity analyses: Within-individual associations of gabapentinoid treatment and adverse outcomes – comparing all time before the first collected medication to all to all time after (n=191,973).

	**All gabapentinoids Incidence Rate Ratio (95% CI)**
Suicidal behavior	1.56 (1.47 to 1.65)
Accidental overdoses	1.77 (1.71 to 1.85)
Head and body injuries	1.25 (1.23 to 1.27)
Transport accidents and offences	0.81 (0.76 to 0.85)
Arrests for violent crime	1.22 (1.16 to 1.29)

Supplemental Table 4. Sensitivity analyses: Within-individual associations of gabapentinoid, pregabalin, and gabapentin treatment and adverse outcomes.

	**All gabapentinoids (n=191,973) HR (95% CI)**	**Pregabalin only (n=120,664) HR (95% CI)**	**Gabapentin only (n=85,360) HR (95% CI)**
**Transport accident treatment**	1.11 (0.99 to 1.25)	1.17 (1.03 to 1.34)	0.96 (0.77 to 1.20)
**Transport offences**	1.04 (0.97 to 1.12)	1.09 (1.00 to 1.17)	0.76 (0.62 to 0.92)
**Head and neck injuries**	1.14 (1.07 to 1.22)	1.20 (1.12 to 1.30)	0.95 (0.84 to 1.08)
**Body injuries**	1.25 (1.21 to 1.28)	1.30 (1.26 to 1.34)	1.09 (1.04 to 1.15)
**Convictions for violent crime**	1.11 (0.99 to 1.25)	1.13 (1.00 to 1.28)	1.00 (0.73 to 1.37)

Supplemental Table 5. Number of events for within-individual associations between gabapentinoid treatment and adverse outcomes by age[a].

	Age 15-24	Age 25-34	Age 35-44	Age 45-54	Age 55-64	Age 65+
**All gabapentinoids**						
Suicidal behavior	5,939	4,691	3,715	2,902	1,174	772
Accidental overdoses	7,254	7,222	6,714	5,911	3,228	2,692
Head and body injuries	11,202	11,910	13,852	13,751	13,691	38,487
Transport accidents and offences	3,262	5,167	5,195	3,437	1,544	1,287
Arrests for violent crime	3,781	4,857	3,717	2,085	490	112
**Pregabalin only**						
Suicidal behavior	5,602	4,350	3,315	2,431	964	587
Accidental overdoses	6,543	6,561	5,895	4,832	2,494	1,714
Head and body injuries	9,615	9,642	10,193	9,127	8,129	20,712
Transport accidents and offences	2,642	4,364	4,125	2,440	938	628
Arrests for violent crime	3,290	4,254	3,046	1,593	336	61
**Gabapentin only**						
Suicidal behavior	695	653	660	652	281	245
Accidental overdoses	1,177	1,314	1,294	1,514	887	1,171
Head and body injuries	2,248	3,053	4,526	5,423	6,296	19,443
Transport accidents and offences	875	1,137	1,332	1,165	672	700
Arrests for violent crime	764	927	867	604	176	56

[a] Number of events by age are reported for Figure 2, Suppl. Figure 1, and Suppl. Figure 2.

Supplemental Table 6. Number of events for adverse outcomes[a].

	**Total number of events**	**Events during non-treatment periods**	**Events during treatment periods**
**All gabapentinoids**			
Suicidal behavior	19,359	11,762	7,759
Accidental overdoses	33,188	20,256	12,932
Head and body injuries	103,649	68,628	35,021
Transport accidents and offences	19,983	15,611	4,372
Arrests for violent crime	15,056	11,071	3,985
**Pregabalin only**			
Suicidal behavior	17,383	10,618	6,765
Accidental overdoses	28,172	17,272	10,900
Head and body injuries	67,886	46,213	21,673
Transport accidents and offences	15,195	11,856	3,339
Arrests for violent crime	12,591	9,150	3,441
**Gabapentin only**			
Suicidal behavior	3,217	2,380	837
Accidental overdoses	7,391	5,361	2,030
Head and body injuries	41,311	28,032	13,279
Transport accidents and offences	5,925	4,909	1,016
Arrests for violent crime	3,397	2,857	540

[a] Number of events by age are reported for Figure 1.

Supplemental Table 7. Gabapentinoid dispenses during the study period.

Number of individuals with a gabapentinoid dispense	272,013
Number of individuals treated* with gabapentinoids	191,937
Number of individuals with a one-off dispense	80,076
Total number of dispenses in the treated cohort	5,061,854
Median (interquartile range) number of dispenses	8 (3, 24)
Median (interquartile range) days of treatment duration	104 (32, 300)

* Treatment is defined as at least two consecutively collected prescriptions within 6 months.

Supplemental Figures 3-7. Age distribution in the gabapentinoid cohort for adverse outcomes

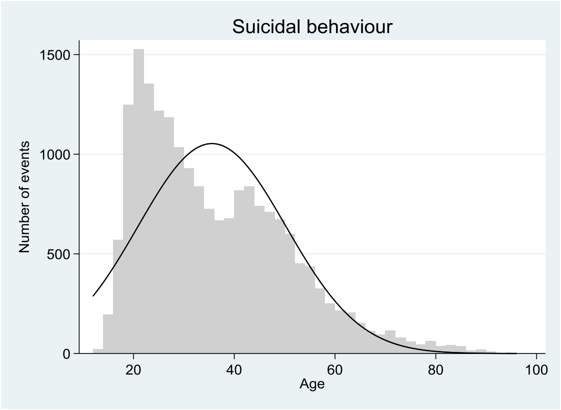

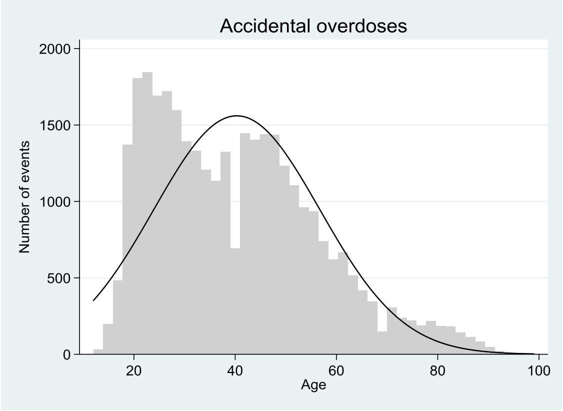

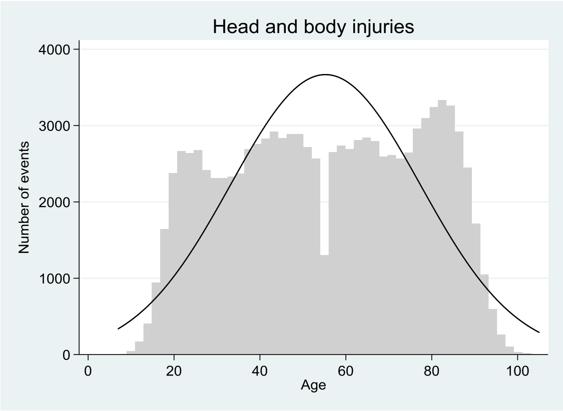
Head and body injuries
Number of events
0
1000
2000
3000
4000
0
20
40
60
80
100
Age

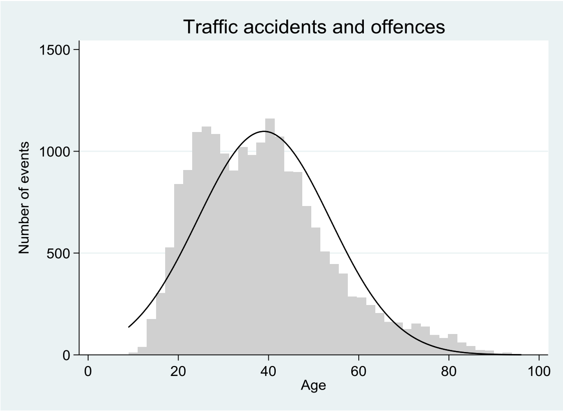
Traffic accidents and offences
Number of events
0
500
1000
1500
0
20
40
60
80
100
Age

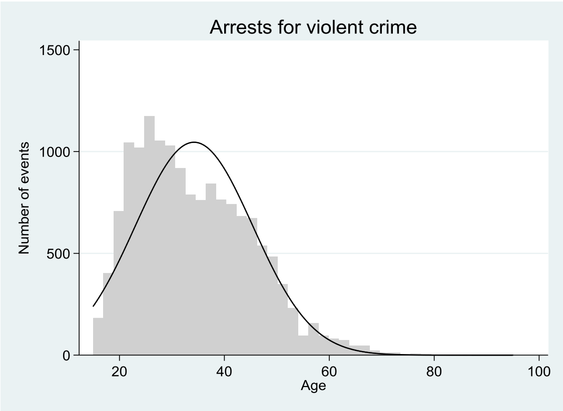
Arrests for violent crime
Number of events
0
500
1000
1500
20
40
60
80
100
Age

Supplemental Figure 8. Age distribution of accidental overdoses in the general population during the study period

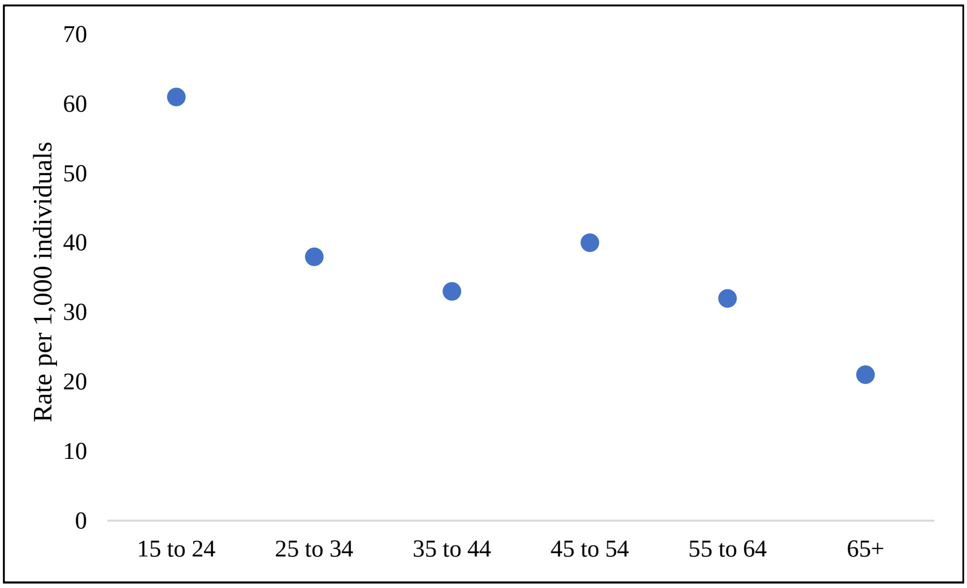

The rate per 1,000 individuals is adjusted for population increase during the study period
